# 贝伐珠单抗治疗非小细胞肺癌所致恶性胸腔积液的研究进展

**DOI:** 10.3779/j.issn.1009-3419.2019.02.07

**Published:** 2019-02-20

**Authors:** 玉杰 刘, 攀文 田

**Affiliations:** 610041 成都，四川大学华西医院呼吸与危重症医学科 Department of Respiratory and Critical Care Medicine, West China Hospital, Sichuan University, Chengdu 610041, China

**Keywords:** 贝伐珠单抗, 肺肿瘤, 恶性胸腔积液, Bevacizumab, Lung neoplasms, Malignant pleural effusion

## Abstract

肺癌是全球范围内发病率最高的恶性肿瘤，晚期肺癌所致的恶性胸腔积液（malignant pleural effusion, MPE）严重影响患者的生活质量和预后。MPE的治疗手段包括胸腔穿刺术、胸膜固定术、胸腔埋管引流、胸腔内灌注治疗等。血管内皮生长因子（vascular endothelial growth factor, VEGF）及其受体是一组影响血管生成的重要配体和受体，是控制血管生成的主要因素，在MPE的形成中发挥重要作用。贝伐珠单抗是一种重组的人源化VEGF单克隆抗体，可与内源性VEGF竞争性结合VEGF受体，抑制新血管生成以及降低血管通透性，阻碍胸腔积液形成，延缓肿瘤发展进程。本综述旨在讨论贝伐珠单抗治疗非小细胞肺癌所致恶性胸腔积液的研究进展，探讨贝伐珠单抗临床应用的方法、疗效、安全性以及未来的发展方向。

## Abstract (en)

Lung cancer is the most commonly diagnosed cancer worldwide. Malignant pleural effusion (MPE) caused by advanced lung cancer seriously affect the patients' quality of life and prognosis. The management of MPE includes thoracentesis, pleurodesis, indwelling pleural catheters and drug perfusion in pleural cavity. Vascular endothelial growth factor (VEGF) and its receptor are a group of important ligands and receptors that affect angiogenesis. They are the main factors controlling angiogenesis, and they play an important role in the formation of MPE. Bevacizumab is a recombinant humanized VEGF monoclonal antibody, competitively binding to endogenous VEGF receptor. Bevacizumab can inhibit new blood vessel formation, reduce vascular permeability, prevent pleural effusion accumulation and slow the growth of cancers. This review aims to discuss the progress of bevacizumab in the treatment of MPE caused by non-small cell lung cancer (NSCLC), and explore the clinical application, efficacy, safety and future direction of bevacizumab.

肺癌是全球范围内发病率最高的恶性肿瘤，其中非小细胞肺癌（non-small cell lung cancer, NSCLC）是肺癌最常见的类型，占肺癌的80%-85%^[1]^。恶性胸腔积液（malignant pleural effusion, MPE）是晚期NSCLC的常见并发症之一，MPE影响患者呼吸功能，导致呼吸困难和发绀等临床症状，严重降低患者生活质量。MPE诊断后患者的中位生存期为3个月-12个月，MPE的发生提示预后不良^[2]^。MPE的治疗原则是在针对原发肿瘤进行全身治疗的基础上，对胸腔进行局部治疗，其治疗策略包括：单纯胸腔穿刺（可反复实施）、胸腔埋管引流、胸膜固定术（通过胸腔镜或胸腔引流管注射胸膜硬化剂，包括滑石粉、强力霉素、四环素、博来霉素等）、胸腔内灌注治疗（化疗药物、免疫调节剂等）^[3-5]^。贝伐珠单抗是VEGF单克隆抗体，通过抑制血管内皮生长因子（vascular endothelial growth factor, VEGF）发挥抗肿瘤作用。近年来有较多研究探索贝伐珠单抗在晚期NSCLC所致MPE中的治疗价值，本文对这一领域的新进展作一综述。

## 1 贝伐珠单抗治疗MPE的机制

MPE的发病机制目前尚未完全明确，可能与免疫效应因子和增加血管通透性的调节因子相关，VEGF通过增加血管通透性在MPE的发生中起着重要作用^[6]^。VEGF是一种内皮生长因子家族，包括VEGF-A、B、C、D、E和胎盘生长因子，其特异性结合VEGF受体（vascular endothelial growth factor receptor, VEGFR），激活丝裂原活化蛋白激酶（mitogen-activated protein kinase, MAPK）通路，三磷酸肌醇激酶（phosphatidylinositol 3-kinase, PI3K）/蛋白激酶B（protein kinase-B, PKB）双通路以及蛋白激酶C（protein kinase-C, PKC）通路等，不同细胞内信号通路的激活，可促进新生血管形成，肿瘤细胞迁移，增加血管的通透性^[2, 6, 7]^。VEGF主要由低氧或其他应激条件下的细胞产生，尤其是在生长和重塑过程中高度表达的细胞，包括：肿瘤、冠状动脉硬化、女性生殖周期中的增殖期的子宫内膜细胞等^[6]^。VEGF通过诱导内皮细胞分化、促使内皮细胞连接的完整性丧失、导致细胞间缝隙形成等多种机制使血管通透性增加^[7]^。VEGF可刺激肿瘤血管内皮细胞，诱导血管形成，提高血管通透性，是MPE形成的重要介质。

贝伐珠单抗是一种重组的人源化VEGF单克隆抗体，通过抑制VEGF发挥抗肿瘤作用，使现有血管退化，抑制新血管生成以及降低血管通透性，阻碍胸腔积液形成，延缓肿瘤发展进程。研究发现该药对晚期NSCLC、结肠癌、直肠癌、乳腺癌、肾癌、宫颈癌及卵巢癌等多种实体肿瘤具有显著的临床疗效^[8]^。针对MPE，贝伐珠单抗也显示出一定的治疗效果。

## 2 贝伐珠单抗治疗晚期NSCLC所致MPE的临床研究

贝伐珠单抗治疗晚期NSCLC所致MPE的给药方法有两种：静脉输注和胸腔内注射。胸腔积液的疗效评价是基于影像学（超声或CT）对胸腔积液量的判断，但其评价标准尚未完全统一。多数研究中，完全缓解（complete response, CR）是指胸腔积液完全消失且持续4周以上，部分缓解（partial response, PR）是指胸腔积液最大深度降低≥50%且持续4周以上。少数研究中，胸腔积液最大深度降低≥30%且持续4周以上即认为达到PR^[9, 10]^。客观缓解率（objective response rate, ORR）是CR和PR患者占全部纳入患者的比值。

### 2.1 贝伐珠单抗静脉输注

多项临床研究证实，静脉输注贝伐珠单抗联合化疗对晚期非鳞状细胞NSCLC伴MPE有较好的临床疗效，可以缓解病情，改善生存质量，延长生存期。Tamiya等^[11]^完成的一项前瞻性、多中心、单臂、开放性2期临床研究发现，23例伴有MPE的NSCLC患者以卡铂、紫杉醇和贝伐珠单抗治疗6周期后，其疾病控制率（disease control rate, DCR）为87.0%，ORR达到91.3%。治疗前血浆中VEGF中位水平为513.6 pg/mL，治疗后VEGF中位水平为25.1 pg/mL，较治疗前明显下降。Usuia等^[4]^进行的一项前瞻性、多中心、单臂、开放性Ⅱ期临床研究发现，28例伴有MPE的NSCLC患者，以卡铂、培美曲塞和贝伐珠单抗治疗6周期，胸腔积液控制率（pleural effusion control rate, PECR）为92.9%，ORR为46.4%。Kitamura等^[12]^回顾性分析了13例具有MPE的NSCLC患者，贝伐珠单抗联合化疗PECR达到92.0%。Masago等^[13]^和Tao等^[14]^的小样本回顾性临床研究也发现，静脉输注贝伐珠单抗联合化疗能有效控制晚期NSCLC所致MPE。上述研究均证实，贝伐珠单抗联合化疗对MPE治疗有效，可以延长患者的生存期。其局限性是，这些研究多为单臂研究，缺乏对照组，而且样本量很小，其研究结果可能存在偏倚。上述临床研究数据汇总见表 1所示。

**1:** 静脉输注贝伐珠单抗联合化疗治疗晚期NSCLC所致MPE Bevacizumab plus chemotherapy using by intravenous infusion in the treatment of MPE caused by advanced NSCLC

Author (year)	Country	Research design	Case	Methods	ORR
Tamiya M (2013)^[11]^	Japan	Prospective, multi-center, single-arm, open-label phase Ⅱ trial	23	Bev (15 mg/kg, every 3 weeks, for 6 cycles)+carboplatin+paclitaxel	91.3%
Kitamura K (2013)^[12]^	Japan	Retrospective, single-center, single-arm	13	Bev (15mg/kg, every 3 weeks, for 6 cycles)+carboplatin	PECR: 92.3%
Masago K (2015)^[13]^	Japan	Retrospective, multi-center, single-arm	21	Bev (15 mg/kg, every 3 weeks) +chemotherapy	71.4%
Chi J (2016)^[9]^	China	Prospective, single-center, randomized-controlled, open-label	46	group 1: Bev (5 mg/kg, every 3 weeks, for 6 cycles)+pemetrexed+carboplatin; group 2: pemetrexed+carboplatin	Group 1: 87.0%; Group 2: 65.2%
Usuia K (2016)^[4]^	Japan	Prospective, multi-center, single-arm, open-label phase Ⅱ trial	28	Bev (15 mg/kg, every 3 weeks, for ≤6 cycles)+pemetrexed+carboplatin	46.4%
Tao H (2017)^[14]^	China	Prospective, single-center, single-arm	21	Bev (15 mg/kg or 7.5 mg/kg, every three weeks, for ≤6 cycles)+chemotherapy	81.0%
Li YL (2018)^[36]^	China	Prospective, single-center, randomized-controlled	86	group 1: Bev (2.5 mL/kg, every 3 weeks)+cisplatin; group 2: cisplatin.	Group 1: 90.7%; Group 2: 79.1%
Bev: bevacizumab; ORR: objective response rates; PECR: pleural effusion control rate; NSCLC: non-small cell lung cancer.					

静脉输注贝伐珠单抗的用药剂量尚不统一。有研究^[15]^显示静脉输注贝伐珠单抗15 mg/kg较7.5 mg/kg能获得更好的ORR、无进展生存期（progression-free survival, PFS）和总生存期（overall survival, OS）。但也有研究^[16]^显示静脉输注7.5 mg/kg和15 mg/kg的贝伐珠单抗治疗，均能显著提高患者的ORR和PFS。两种用药剂量对NSCLC的疗效没有明显差异，而高剂量的贝伐珠单抗导致更多的不良事件^[17-19]^。目前美国和中国批准贝伐珠单抗的剂量均为15 mg/kg，欧盟批准的剂量为15 mg/kg和7.5 mg/kg^[20, 21]^。

### 2.2 贝伐珠单抗胸腔内注射

早在2013年，Du等^[22]^报道了一项前瞻性、单中心、随机对照研究，共纳入了70例晚期NSCLC伴有MPE的患者，随机分为两组，在胸腔置管引流后接受顺铂联合贝伐珠单抗胸腔内注射治疗或胸腔内顺铂单药化疗，每2周给药1次，共3个治疗周期。结果发现，两组ORR分别为83.3%和50.0%（*P* < 0.05）。联合贝伐珠单抗治疗组患者胸水VEGF水平在治疗后显著下降，在治疗前胸水VEGF高水平的患者中尤为有效。

在此研究之后，多项临床研究^[10, 22-34]^探索了胸腔内注射贝伐珠单抗对晚期NSCLC伴MPE的临床疗效和安全性，数据汇总见表 2。Chen等^[30]^进行了一项单中心的回顾性临床研究，共纳入574例具有MPE的NSCLC患者，其治疗方案分为四组：胸腔注射贝伐珠单抗、胸腔内注射化疗药物、胸腔内注射免疫调节剂和单纯胸腔穿刺术，四组患者胸腔积液的控制率分别为90.00%、82.40%、67.59%和46.55%。胸腔内注射贝伐珠单抗治疗MPE的控制率明显高于其他三种治疗方法。一项*meta*分析汇总了769例伴有MPE的NSCLC患者，胸腔内注射贝伐珠单抗联合铂类药物与单纯铂类药物治疗相比，联合贝伐珠单抗治疗组患者的胸腔积液明显减少，胸痛、呼吸困难等临床症状明显缓解，治疗后胸腔积液中VEGF水平明显降低^[35]^。

**2:** 胸腔内注射贝伐珠单抗治疗晚期NSCLC所致MPE Bevacizumab using by intrapleural infusion in the treatment of MPE caused by advanced NSCLC

Author (year)	Country	Study design	Case	Methods	Duration of drainage	ORR
Nan Du (2013)^[22]^	China	Prospective, single-center, randomized-controlled	70	Group 1: Bev (300 mg, every 2 weeks, for 3 cycles)+cisplatin; group 2: cisplatin	Not mentioned	Group 1: 83.3% Group 2: 50.0%
Han N (2013)^[10]^	China	Prospective, single-center, randomized-controlled	42	Group 1: Bev (5 mg/kg, every 3 weeks, for 4 cycles)+cisplatin+pemetrexed; group 2: cisplatin+pemetrexed	2 d-3 d	Group 1: 85.0%; Group 2: 68.2%
Kang HR (2014)^[23]^	China	Retrospective, single-center, randomized-controlled	34	Group 1: Bev (200 mg, every 1 week, for 4 cycles)+cisplatin; group 2: cisplatin	Not mentioned	Group 1: 85.0%; Group 2: 57.5%
Qu B (2015)^[24]^	China	Prospective, single-center, randomized-controlled	63	Group 1: Bev (5 mg/kg, every 1 week, for 3 cycles)+cisplatin; group 2: cisplatin	2 d-3 d	Group 1: 84.3%; Group 2: 61.3%
Chen L (2015)^[25]^	China	Prospective, single-center, randomized-controlled	54	Group 1: Bev (5 mg/kg, every 3 weeks, for 2 cycles)+cisplatin; group 2: cisplatin	Not mentioned	Group 1: 85.7%; Group 2: 69.2%
Yan YH (2015)^[26]^	China	Prospective, single-center, randomized-controlled	92	Group 1: Bev (300 mg/m^2^, every 1 week); group 2: cisplatin	1 d-4 d	Group 1: 84.78%; Group 2: 56.52%
Nan Q (2016)^[27]^	China	Prospective, single-center, randomized-controlled	24	Group 1: Bev (5 mg/kg, every 3weeks, for 4 cycles)+paclitaxel; group 2: paclitaxel	Not mentioned	Group 1: 78.60%; Group 2: 50.00%
Lin H (2016)^[28]^	China	Prospective, single-center, randomized-controlled	94	Group 1: Bev (5 mg/kg, every week, for 3 cycles)+cisplatin; group 2: cisplatin	2 d-3 d	Group 1: 70.2%; Group 2: 44.7%
Liu HP (2016)^[29]^	China	Prospective, single-center, randomized-controlled	84	Group 1: Bev (5 mg/kg, every 3weeks, for 4 cycles)+pemetrexed+cisplatin; group 2: pemetrexed+cisplatin	2 d-3 d	Group 1: 83.33%; Group 2: 64.29%
Chen DW (2017)^[30]^	China	Retrospective, single-center, randomized-controlled	574	Group 1: Bev (100 mg or 200 mg, every week until a response); group 2: chemotherapy; group 3: biological response modifiers; group 4: simple puncture to drain the effusion	Not mentioned	Group 1: 90.00%; Group 2: 82.40%; Group 3: 67.59%; Group 4: 46.55%
Jiang M (2017)^[31]^	China	Prospective, single-center, randomized-controlled	52	Group 1: Bev (5 mg/kg, every 1 week, for 3 cycles)+carboplatin; group 2: carboplatin	2 d-4 d	Group 1: 87. 5%; Group 2: 60.7%
Xue DF (2017)^[32]^	China	Prospective, single-center, randomized-controlled	82	Group 1: Bev (5 mg/kg, every 1 week, for 3 cycles)+cisplatin; group 2: cisplatin	Not mentioned	Group 1: 92.68%; Group 2: 75.61%
Zhao JZ (2018)^[33]^	China	Prospective, single-center, randomized-controlled	48	Group 1: Bev (200 mg, every 1 week, for 8 cycles)+carboplatin; group 2: Bev (200 mg, every 1 week, for 8 cycles)	Not mentioned	Group 1: 83.3%; Group 2: 55.6%
Sun ZJ (2018)^[37]^	China	Prospective, single-center, randomized-controlled	48	Group 1: Bev (5 mg/kg, every 3 weeks, for 4 cycles)+gemcitabine; group 2: gemcitabine	Not mentioned	Group 1: 83.3%; Group 2: 40.9%

上述研究均表明，胸腔内注射贝伐珠单抗能有效控制晚期NSCLC所致MPE，缓解呼吸困难、发绀等症状，提高患者生活质量，延长生存期。但是，这些研究多为小样本的临床研究，或为回顾性临床研究，存在一定偏倚。且胸腔内注射贝伐珠单抗是一种颇有争议的治疗方式，在治疗过程中，其不确定因素（给药剂量、间隔时间、给药次数）较多。因此，其研究结论需客观解读。

在大多数研究中胸腔注射贝伐珠单抗的剂量为100 mg/次-300 mg/次，也有研究单次给药剂量为5 mg/kg。有研究发现低剂量（不高于100 mg/次）的贝伐珠单抗和高剂量（高于100 mg/次）的贝伐珠单抗相比，患者PFS和OS差异没有统计学意义，低剂量贝伐珠单抗组的不良事件发生率和程度明显低于高剂量组^[35]^。多数研究中，胸腔内给药的间隔时间为每1周-3周给药1次，总共给药次数为2次-4次。目前尚没有临床研究比较贝伐珠单抗静脉输注和胸腔内注射两种不同给药方式治疗MPE的临床疗效和安全性，未来需要开展前瞻性临床对照研究来进一步探索。

## 3 贝伐珠单抗治疗晚期NSCLC所致MPE的安全性

多项临床研究证实静脉输注贝伐珠单抗治疗晚期NSCLC所致的MPE安全有效，主要不良事件为血液系统副作用，包括中性粒细胞减少、血红蛋白降低、血小板减少等。其他的不良事件还包括神经系统反应、厌食、高血压、蛋白尿等。目前尚没有药物导致死亡事件，没有严重的出血和其他严重毒性反应^[11, 12, 14]^。

胸腔内注射贝伐珠单抗出现的不良反应多为1级-2级骨髓毒性。有研究发现贝伐珠单抗组高血压的发生率高于局部化疗组，但贝伐珠单抗相关的高血压仅为1级-2级，没有出现3级-4级的严重不良反应^[22, 27]^。

## 4 尚需解决的问题

### 4.1 给药方式、剂量和疗程

贝伐珠单抗的给药方式仍存在争议。在贝伐珠单抗的说明书中，其唯一的给药方式为静脉输注。虽然国内有较多临床研究报道了贝伐珠单抗胸腔注射治疗MPE的有效性，但在临床实践中，胸腔注射贝伐珠单抗仍应谨慎。在采用这种给药方式治疗前应进行充分的知情沟通，获患方同意后方可用药。此外，胸腔内注射贝伐珠单抗的剂量目前没有统一标准，胸腔内给药是贝伐珠单抗单药还是与铂类药物联用尚无共识。不仅如此，胸腔内给药的次数也存在不确定因素，部分患者经治疗后胸腔积液减少，后续再次行胸腔穿刺或胸腔置管的风险增大，如何按照计划完成胸腔内给药疗程是对临床医生的挑战。

### 4.2 疗效评价指标和工具

NSCLC患者的治疗疗效通过实体瘤疗效评价标准（Response Evaluation Criteria in Solid Tumors, RECISTs）标准进行评价，按照RECISTs标准，胸腔积液属于不可测量病灶。针对不可测量病灶如何进行定量评估，目前尚无统一标准。但统一的疗效评价标准对开展高质量的临床研究至关重要。既往研究中对于胸腔积液的测量多采用超声检查，超声检查虽然简便易行，但超声对胸腔积液量的判断容易受到患者检查时的体位影响。因此，需要有更加客观且干扰因素少的诊断工具来定量评价胸腔积液。

## 5 未来展望

MPE的发病机制颇为复杂，VEGF信号通路在一定程度上揭示了MPE的形成机制，针对VEGF通路不同环节的药物为MPE的治疗带来希望。贝伐珠单抗无论联合化疗静脉输注，还是胸腔内给药，都可以缓解晚期NSCLC所致MPE的压迫症状，提高生活质量，延长生存期，不良反应也在可接受范围之内。目前缺乏高质量的随机对照临床研究来对比静脉给药和胸腔给药的疗效和安全性的差异。胸腔内给药剂量和疗程缺乏统一标准，准确定量评价胸腔积液仍存在挑战，未来需要开展大样本多中心的临床研究来进一步证实。
